# Achievement of LDL-Cholesterol Goals in Patients Receiving LLT in Primary Care: TERESA-AP Study: LDL-Cholesterol Goals in Primary Care

**DOI:** 10.1155/2024/4227941

**Published:** 2024-07-03

**Authors:** Sergio Cinza-Sanjurjo, Vivencio Barrios, David Fierro-González, Jose Polo-García, Vicente Pallarés-Carratalá

**Affiliations:** ^1^ Milladoiro Health Centre Santiago de Compostela Health Area, Santiago de Compostela, Spain; ^2^ Santiago de Compostela Research Institute (IDIS), Santiago de Compostela, Spain; ^3^ Networking Biomedical Research Center-Cardiovascular Diseases (CIBER-CV), Madrid, Spain; ^4^ Medicine Department Santiago de Compostela University, San Francisco Street 15701, Santiago de Compostela, Spain; ^5^ Cardiology Department, H Ramón y Cajal 28034, Madrid, Spain; ^6^ Department of Medicine University of Alcala 28801, Madrid, Spain; ^7^ Armunia Health Centre, León, Spain; ^8^ Casar de Cáceres Health Centre, Cáceres, Spain; ^9^ Department of Medicine Universitat Jaume I 12071, Castellón, Spain

**Keywords:** cardiovascular risk, LDL-cholesterol goals, lipid-lowering therapy, primary care

## Abstract

**Background and Aim:** Since 2019, LDL-cholesterol (LDL-C) is the risk factor with the strictest goals and the most difficult to reach, due to its role in the development of atherosclerotic plaque and, therefore, cardiovascular risk. The objective of the TERESA-AP study is to analyze the degree of LDL-C control in patients followed up in primary care with lipid-lowering drug treatment (LLT).

**Methods:** Observational, multicenter, cross-sectional, nationwide study was conducted, in which 50 PC physicians recruited 929 patients who were receiving LLT during at least the preceding 6 months. The variables required to estimate the patients' cardiovascular risk and LDL control were recorded.

**Results:** Nearly half of sample was women (50.5%), and the mean age was 67.8 (10.4) years. High blood pressure (65.3%) and sedentary lifestyle (59.7%) were the most frequent risk factors. Recommended goals were reached in 26.0% (95% CI: 23.3%–29.0%) of patients, with a slightly higher percentage in patients with cardiovascular disease (CVD) (26.7%), diabetes mellitus (DM) (35.5%), and a lower one in patients with chronic kidney disease (CKD) (12.1%). The most frequent drug treatments were statin monotherapy (69.0%) and statin with ezetimibe combination (27.6%), with moderate-intensity statins being the most commonly used in both groups.

**Conclusions:** On average, only a quarter of the patients followed up in PC and who receive drug treatment reach their therapeutic targets. This percentage is slightly higher if the patients have CVD and DM and lower if they have CKD. The most commonly used therapeutic strategy is moderate-intensity statins, both in monotherapy and in combination with ezetimibe.

## 1. Introduction

Mortality from cardiovascular disease (CVD) has shown a downward trend in recent decades in Western countries; however, it is currently still the leading cause ahead of cancer [1]. The pathophysiological mechanism of CVD is based on the cardiovascular continuum, according to which the presence of different cardiovascular risk factors (CVRFs), together with a poor control of these, first favours the development of subclinical damage in the target organs (TODs), such as albuminuria or left ventricular hypertrophy, which will progress into CVD [2].

The approach to cardiovascular risk (CVR) in our patients includes a series of strategies: modifying lifestyles (physical activity of the patient [3], implementing a Mediterranean diet [3, 4], and smoking cessation [3]) and controlling CVRF (hypertension [3, 5], hypercholesterolemia [3, 6], and diabetes mellitus (DM) [7]), whose goals and strategies will vary depending on the estimated CVR of each patient, as well as other clinical factors such as age or comorbidities.

In order to know the CVR of each patient, there are tools that depend on epidemiological and clinical variables and allow us to estimate such risks. The most widely used tool is the recently updated *Systematic COronary Risk Evaluation* project (SCORE2) [8, 9]. In patients with a higher CVR, a poor control of CVRF or failure to correct CVRF is associated with an increased risk of cardiovascular events. In Spain, recent data indicate that the control of these CVRFs is low: 19.8% of patients are active smokers, the degree of control of hypertension is 52.1%, DM is 68.4%, and only 34.0% of patients with hypercholesterolemia have a good control of LDL-cholesterol (LDL-C) [10].

The control of LDL-C levels seems to be the most important challenge in clinical practice, since the *European Guidelines for the Approach to Dyslipidemia* (ESC2019) modified the therapeutic targets following published evidence in clinical trials with iPCSK9 that showed a better prognosis with lower LDL levels [6]. The results on degree of control have been published recently with these new targets. However, most of them have little external validity as they were obtained in hospital settings or in patients after acute coronary syndrome. Therefore, there is virtually no evidence of achievement of goals in patients followed up in primary care (PC) who are undergoing treatment. We proposed the TERESA-AP study (achievement of therapeutic targets in patients with statins alone or in combination with ezetimibe in PC) with the main objective of knowing the degree of control of LDL-C, taking into account the ESC2019 [6], in patients receiving drug treatment and followed up in PC.

## 2. Material and Methods

The protocol of this study was presented to the CREC San Carlos, Madrid, and approved on January 7, 2022, with the code 21/786-O_M_No SP.

### 2.1. Study Design

We conducted a nationwide, observational, cross-sectional, multicenter study, in which 50 investigators participated and PC physicians from the National Health System (SNS) who recruited 929 valid patients in 6 months (from November 2, 2022, to May 31, 2023).

### 2.2. Patients

The sample size was estimated considering an infinite target population, recruited by simple random sampling, taking into account a degree of control of hypercholesterolemia of 34.0% (10), a 95% confidence level, and an accuracy of 3.25%, and assuming that 10% of the sample had to be excluded due to nonvalid data. Thus, the estimated sample was 880 patients.

Adult patients codified with diagnosis of hypercholesterolemia who had been receiving drug treatment for at least the last 6 months and who had evidence of adherence by at least one of the two proposed methods were included: the Morisky–Green test [11] or collecting their electronic prescription medication at the pharmacy, which has been validated for adherence to treatment of patients with dyslipidemia [6]. Patients with functional impairment or severe mental or cognitive illness and those who, in the opinion of the investigator, cannot be adequately followed up in the chronic care program in routine clinical practice were excluded.

### 2.3. Variables

All the data were obtained from the information included in the medical records: epidemiological variables (age and sex) and the clinical history necessary to estimate CVR by SCORE2 [8, 9], as well as the drug treatments for LDL-C control and the physical examination and lab tests that allow assessing the achievement of therapeutic targets. Also, the previous treatments, if any, were recorded. The most recent blood tests with that treatment, the cause that motivated the change of treatment, and the presence of side effects with both current and previous treatments were also recorded.

All the information from each patient was recorded in coded form in an eCRF, with individual access for each investigator. The recorded data were submitted to internal validation programs that automatically generate discrepancy alerts that must be reassessed by the researcher to confirm whether the data is correct (four patients were eliminated from the final analysis due to lack of data quality: one due to absence of the date of the informed consent and three patients due to the lack of blood tests in the last 6 months).

### 2.4. Statistical Analysis

The descriptive statistical analysis of the variables was carried out using the mean, standard deviation (SD), median, interquartile range (IQR), and calculation of proportions. The bivariate analysis was performed using the *χ*^2^ test, Student's *t*-test, and ANOVA tests or their counterpart nonparametric tests when the data do not follow a normal distribution (Mann–Whitney *U* or Kruskal–Wallis).

The CVR was estimated using SCORE2 [8] and SCORE-OP in patients over 70 years of age [9], and the therapeutic targets for each risk level were those defined in the ESC2019 [6].

All results are presented with the mean and the 95% confidence interval (95% CI) and will be expressed to one decimal place, although during the calculation, no rounding will be applied in any case. A *p* value of less than 0.05 was considered statistically significant. For data processing and analysis, the statistical package SPSS 22.0 for Windows will be used.

## 3. Results

### 3.1. Sample Description

A total of 933 patients were included, of which 929 patients were valid for the final analysis. All the patients met the adherence criterion in at least one of the two methods: 79.7% was adherent according to the Morisky–Green test and 97.7% according to the control of medication discontinuation.

Table 1 shows the clinical and epidemiological characteristics of the sample, with a proportional balance between sexes (50.5%, women) and a mean age of 67.8 (10.4) years. The most frequent CVRFs were hypertension (65.3%) and sedentary lifestyle (59.7%). CVD was present in 21% of patients, with ischemic heart disease being the most frequent (11.0%). The CVR estimated by SCORE2 was high or very high in 78.8% of patients.

### 3.2. Achievement of LDL-C Goals

**Figure 1:**
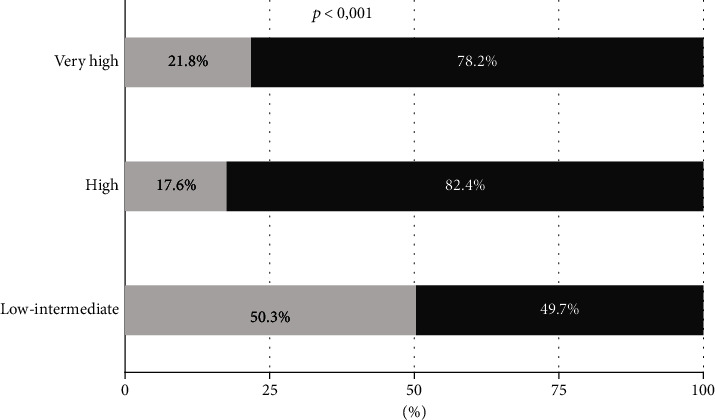
Achievement of goals according to cardiovascular risk.

Recommended goals in the CPGs were reached in 26.0% (95% CI: 23.3%–29.0%) of patients. Patients with lower risk achieve the goals the most (50.3%), while patients with high (17.6%) and very high risk (21.8%) have a lower achievement of these goals (*p* < 0.001) (Figure 1).

**Figure 2:**
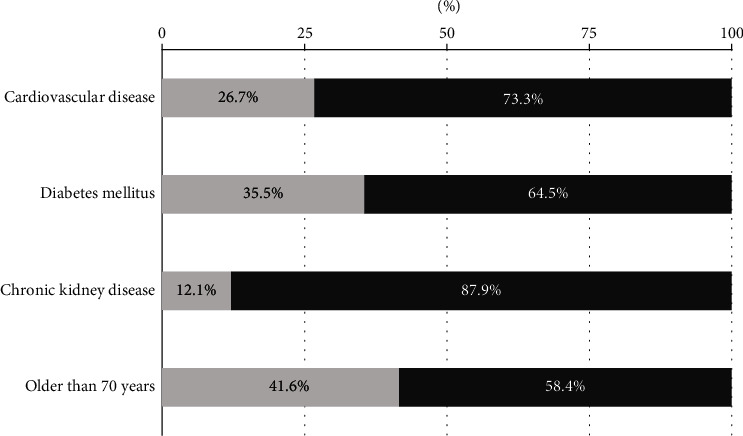
Achievement of goals according to disease determinants.

The presence of disease determinants reflects a different degree of control, as can be seen in Figure 2. Only 26.7% of patients with CVD achieve LDL-C goals. This degree of control is slightly higher in patients with DM (35.5%) and in patients over 70 years of age (41.6%). Patients with CKD have the worst degree of goal achievement (12.1%).

### 3.3. Current Drug Treatment and Goal Achievement

The most frequently used drug treatment was statin monotherapy (69.0%) and statins with ezetimibe combination therapy (27.6%). Fibrates, alone or in combination, were used in 5.3% and ezetimibe monotherapy in 3.1%. Two-thirds (69.5%) of patients had been receiving treatment for more than 1 year. The PCSK9 inhibitors were prescribed only in one patient.

The most commonly used therapeutic strategy was moderate-intensity statins monotherapy (58.7%) and statins with ezetimibe combination (17.2%), followed by high-intensity statins in combination with ezetimibe (11.2%) and in monotherapy (8.5%).  Table 2 shows the statins and their doses used in monotherapy and in combination with ezetimibe.

According to the CVR of the patients, the most commonly used therapeutic strategy, at all levels of risk, was moderate-intensity statins and their combination with ezetimibe (Table 3). Higher intensity LDL-C-lowering strategies (intermediate- or high-intensity statins, in combination with ezetimibe, or high-intensity statins) are more commonly used in high- and very-high-risk patients.

The most effective therapeutic strategies for reaching the goals were ezetimibe combination with high-intensity statins (45.0%) and with moderate-intensity statins (39.9%), compared to high-intensity statin monotherapy (26.9%) and moderate-intensity statin monotherapy (19.8%) (*p* < 0.001).

### 3.4. Previous Pharmacological Treatment

Among the patients who had not previously received any lipid-lowering drug treatment (58.2%), the most commonly used strategy was moderate-intensity statin monotherapy (73.0%). High-intensity statin monotherapy (7.8%) and combination of both with ezetimibe (7.8% and 3.8%, respectively) were used more rarely.

Other lipid-lowering therapies were used previously in the 41.8% of patients: statins (39.5%) and ezetimibe (2.3%) in monotherapy or in combination.

Changes in therapy for failure to achieve therapeutic targets (70.4%) implied an increase in intensity in 49.0% of patients, whereas in 42.7%, they did not imply a change in intensity; the rest (8.4%) were changes to lower intensity strategies.  Table 4 shows the treatment changes made to strategies involving statins. In spite of this, the treatments currently received by the patients resulted in a reduction of LDL-C compared to baseline values (Table 5).

### 3.5. Presence of Side Effects

The presence of side effects accounted for 13.9% of treatment changes, myalgia (60.0%), and elevated transaminases (22.5%) being the most frequent.

In myalgias, the most frequent treatment change strategies were statin switching (45.8%), discontinuing treatment with statins (29.2%), and lowering the dose of the same statin in monotherapy (20.8%) or combined with ezetimibe (4.2%).

## 4. Discussion

The results of the TERESA-AP study, in a large sample of patients on drug treatment in PC, show that only a quarter of the patients with hypercholesterolemia who are receiving treatment reach the LDL-C goals described by clinical practice guidelines, based on their CVR. The worst group of patients was with chronic kidney disease who achieved objectives only 12.1%. The patients with CVD (26.7%) and DM (35.5%) achieved most frequently the objectives. The majority of patients followed up in PC have a high CVR, and in order to achieve therapeutic targets, they require more intensive lipid-lowering strategies than moderate-intensity statins, which are mostly used at all risk levels. In spite of this, the analysis of treatment changes shows that there is a tendency to change towards higher intensity strategies, which allows us to affirm that, slowly but progressively, physicians are internalizing the new therapeutic targets and the necessary use of other strategies. Another factor that limits the change of treatment could be side effects, present only in 1 in 10 patients, and most of them are of a subjective nature such as myalgias.

After an extensive literature review, our study is the first to be carried out exclusively in PC, which classifies the level of CVR of patients using SCORE2 and considers the current therapeutic goals described in the ESC2019 guidelines. Among the most recent studies, the DA VINCI study also uses the 2019 goals, but in patients who were previously on treatment, so it allows us to really assess the impact of the guidelines. In this study, 33% of the patients achieved the therapeutic goals, a higher figure than ours (26.0%), although they established the CVR using SCORE [12]. This lower degree of control could be explained by the fact that, in our sample, the use of moderate-intensity statin monotherapy is much greater, despite the fact that there is a greater use of combination therapies with ezetimibe (27.6% vs. 9%) [12].

The SANTORINI study presents the most current data, between 2020 and 2021. Ray et al. described a similar degree of control in LDL-C (26.4%) in a very similar sample considering the CVR with 29.2% of the patients with high risk and 70.8% very high risk (in our study, 78.8% of the patients were classified in one of both groups). Compared with our design, all of our patients were taking low-lipid drugs and 69.0% received statin monotherapy, and in the SANTORINI study, 21.8% of the patients did not receive any type of drug treatment and 54.3% received statin monotherapy. In the SANTORINI study, 24.0% of patients received combination therapy [13], like in our study (27.6%). As a lesson from the results discussed, we must highlight that combination therapy should be the cornerstone of the drug treatment of patients with high and very high CVR in order to achieve the therapeutic goals described in ESC2019.

The TERESA study, from the same program to which our study belongs, also included high- and very-high-risk patients, and all of them were taking atorvastatin or rosuvastatin, combined or not with ezetimibe. Here, the degree of control was 31.1%. The high use of statin combination (62.1%) justifies the greater compliance with goals compared to our study [14]. As we have observed, strategies that include high-intensity statins with ezetimibe are those that allow more patients to achieve the recommended goals.

Meeting LDL-C goals only makes sense if we take into account patients' CVR, because the goals are different. Comparing our results with the DA VINCI study, only patients with very high CVR (21.8% vs. 11%) and CVD (26.7% vs. 18%) achieved the goals in a higher proportion, while patients with low-moderate (50.3%) and high CVR (17.6%) complied with the recommendations to a lesser extent than in the DA VINCI study (60% and 25%, respectively) [12]. This is probably because the use of high-intensity statins and combination therapies was higher in our study in patients with very high CVR (10.6% and 38.7%, respectively, in our study, compared to 9% and 42% in the DA VINCI study) and moderate-intensity statin monotherapy was the most commonly used strategy in the rest of the patients.

In addition to patients with CVD, other interesting group in achieving the LDL-C goals is the DM patients. The EPHESUS study analyzed the degree of control in these patients. Başaran et al. described that there are 2.2% of patients without CVD and 8.8% of patients with CVD [15]. Our DM patients achieved in higher proportion (35.5%), maybe it was possible because only half of patients in the EPHESUS patients were taking treatment.

In the last decade, there has been discussion about the need to maintain statin treatment in older patients, despite the best prognosis described [16, 17] and the same safety observed [18]. Our results confirm that this group of patients can achieve a high control with more than 40%.

After publication of the current recommended goals, the degree of compliance was halved, as shown in the same study [19]. Really, the low degree of control in LDL-C is caused by the low use of high-intensity statins and combination therapies that are the best strategies to increase the degree of control. Compared with the DA VINCI study, developed immediately after the publication of the current recommended goals, our results obtained four years after its publication shows that there is an increase in the use of intensive therapies, as in neighboring countries [20, 21]. In view of our results that confirm the results showed in other studies, we have to change our clinical practice prioritizing treatments with high-intensity statin and as a best practice its combination with ezetimibe, to reach the LDL-C objectives [22].

In the lipid-lowering therapies, there is an important therapeutic inertia. The results as TERESA-opinion showed that the physicians identified the determinants that increased CVR, and they also made a correct selection of the most powerful therapeutic strategies, but they estimated that approximately half of their patients had a correct control of LDL-C [23]. As a best control in patients with CVD in our study, DA VINCI, SANTORINI, or EPEHSUS studies confirm that we identify correctly the patients with high CVR. Possibly, the best strategy is to begin with fixed combinations of statins with ezetimibe in patients high and very high CVR, avoiding therapeutic steps of progressive intensification [24, 25]. This strategy will allow greater adherence to therapeutic recommendations in high-risk patients [26, 27], including patients with kidney disease [28], who showed the worst control in our study.

Another justification is the fear of iatrogenesis in the patient when prescribing statins, since the suspicion of side effects is the main cause of dose reduction or discontinuation of statins in patients with high CVR [29]. The observational studies described a higher incidence of side effects associated with statins (17%) compared to clinical trials (4.9%) [30]. The SAMSON study analyzed the identification of these symptoms in patients taking statins, and the authors observed that physicians overestimate the presence of side effects, assuring that they are present in between 10% and 12% of patients undergoing treatment, when the real presence was in 7% [31]. In general, it is estimated that the use of statins increases the risk of adverse effects by 3%–8% depending on the type of statin used [32], so they can be considered very safe treatments [33].

All these arguments could justify the little intensification of treatment in patients who do not reach the goals, which is estimated at 17% for patients with high CVR [34]. This means that many patients do not improve their prognosis, since model simulations estimate that a correct treatment intensification would allow these objectives to be achieved in 57% of patients without CVD and in 42% of patients with CVD [35].

Although our results are solid, obtained from a large sample of PC patients and in line with the most current literature, they are not without limitations. On the one hand, the retrospective and cross-sectional design of the study itself makes us dependent on the information recorded in the medical history and prevents us from establishing causal relationships. The information used for this work is normally used in clinical practice, so it is usually recorded in the history; proof of this is that only four patients were eliminated due to insufficient quality of the data, as explained above. The association established between lower intensity strategies and the low degree of LDL-C control is not definitive and requires longitudinal studies; however, it coincides with the data presented in other contemporary studies such as TERESA, Da VINCI, or SANTORINI, already discussed.

Another interesting aspect is that our sample includes patients on active drug treatment, which could underestimate cases of statin intolerance, despite the fact that our work is not limited to this therapeutic group. From our point of view, the drugs used coincide with what has been published in other contemporary studies, with less than 4% of patients being treated with nonstatin drugs. Therefore, if this is a limitation, it is similar to the rest of studies.

In any case, these limitations do not weaken the credibility of our results and do not limit our objective, which was to describe the degree of LDL-C control in PC. We must be aware that the conclusions are only applicable to patients who are receiving treatment with statins.

## 5. Conclusions

From the above, we can conclude that only a quarter of patients who receive pharmacological treatment in PC achieve the therapeutic LDL-C goals. Most patients are high and very high risk and are the least successful in achieving therapeutic goals, especially patients with CKD and CVD. A possible cause is the high use of moderate-intensity statin monotherapy and the low use of combinations with statins, without treatment intensification despite the poor control of patients.

## Figures and Tables

**Table 1:** Clinical and epidemiological characteristics of the patients in the sample.

	**N**	**Percentage**
Women	469	50.5%
Age, mean (SD)	929	67.8 (10.4)
Cardiovascular risk factors		
High blood pressure	607	65.3%
Diabetes mellitus	330	35.5%
Familial hypercholesterolemia	26	2.8%
Family history of early CVD	102	11.0%
Physical activity		
None	150	16.1%
< 150 min/week	405	43.6%
≥ 150 min/week at fast pace	361	38.9%
Sport/competition activity	13	1.4%
Smoking		
Never	491	52.9%
Smoker	157	16.9%
Former smoker	281	30.2%
Alcohol consumption		
None	571	61.5%
< 40 g (men) and 30 g (women)/day	318	34.2%
≥ 40 g (men) and 30 g (women)/day	40	4.3%
Hypertension-mediated subclinical damage		
Left ventricular hypertrophy	65	7.0%
Retinopathy	21	2.3%
Neuropathy	22	2.4%
Cardiovascular disease	195	21.0%
Ischemic heart disease	102	11.0%
1 episode	98	89.9%
2 episodes	5	4.6%
3 or more episodes	3	2.7%
Atrial fibrillation	65	7.0%
Cerebrovascular disease	64	6.9%
1 episode	57	89.1%
2 episodes	4	6.3%
Peripheral artery disease	39	4.2%
Kidney disease		
eGFR < 60 ml/min/1.73 m^2^	82	8.8%
Albumin/creatinine ≥ 30 mg/g	49	5.3%
Cardiovascular risk (SCORE2)		
Low moderate	197	21.2%
High	393	42.3%
Very high	339	36.5%

**Table 2:** Types of statins and doses used in monotherapy and in combination with ezetimibe.

**Statin and dose**	**%**
Monotherapy	
Simvastatin 20 mg	20.3%
Atorvastatin 20 mg	16.5%
Atorvastatin 40 mg	10.8%
Atorvastatin 10 mg	9.5%
Rosuvastatin 10 mg	8.6%
Rosuvastatin 20 mg	7.6%
Simvastatin 10 mg	6.9%
Simvastatin 40 mg	4.4%
Rosuvastatin 5 mg	3.6%
Atorvastatin 80 mg	3.3%
Pravastatin 40 mg	2.0%
Pitavastatin 2 mg	1.7%
Atorvastatin 30 mg	1.2%
Rosuvastatin 40 mg	0.9%
Atorvastatin 60 mg	0.6%
Pitavastatin 4 mg	0.6%
Pitavastatin 1 mg	0.5%
Pravastatin 20 mg	0.5%
Pravastatin 10 mg	0.3%
Fluvastatin prolib 80 mg	0.2%
Combination therapy with ezetimibe	
Rosuvastatin 20 mg	33.2%
Rosuvastatin 10 mg	31.3%
Atorvastatin 40 mg	12.1%
Atorvastatin 20 mg	7.4%
Atorvastatin 80 mg	6.3%
Simvastatin 20 mg	3.9%
Simvastatin 40 mg	2.0%
Atorvastatin 10 mg	0.8%
Rosuvastatin 40 mg	0.8%
Rosuvastatin 5 mg	0.8%
Pitavastatin 2 mg	0.4%
Pitavastatin 4 mg	0.4%
Pravastatin 20 mg	0.4%
Pravastatin 40 mg	0.4%

**Table 3:** Therapeutic strategies used at each level of risk.

	**Low-moderate CVR**	**High CVR**	**Very high CVR**
High-intensity statin + ezetimibe	6.1%	7.9%	18.0%
High-intensity statin monotherapy	9.1%	6.4%	10.6%
Moderate-intensity statin + ezetimibe	14.7%	15.5%	20.7%
Moderate-intensity statin monotherapy	66.0%	65.1%	46.9%
Low-intensity statin + ezetimibe	0.0%	0.3%	0.0%
Low-intensity statin monotherapy	1.0%	0.3%	0.6%
Ezetimibe monotherapy	1.0%	2.8%	2.1%
Others	2.0%	1.8%	1.2%

**Table 4:** Changes in therapeutic strategy in patients who did not achieve therapeutic goals.

**Previous treatment**	**Current treatment**				
	**Moderate-intensity statin**	**High-intensity statin**	**Low-intensity statin + ezetimibe**	**Moderate-intensity statin + ezetimibe**	**High-intensity statin + ezetimibe**
Low-intensity statin	2.8%	0.3%		0.7%	
Moderate-intensity statin	39.0%	9.0%	0.3%	18.4%	8.4%
High-intensity statin	1.7%			0.3%	4.5%
Low-intensity statin + ezetimibe					0.3%
Moderate-intensity statin + ezetimibe	0.3%			0.3%	0.7%
High-intensity statin + ezetimibe					1.0%

**Table 5:** Blood test parameters with current treatment and previous treatment.

	**Previous test**	**Current test**
Glycemia (mg/dl)	107.9 (38.3)	106.4 (29)
HbA1c (%)	6.4 (1.3)	6.3 (1.1)
Total cholesterol (mg/dl)	210.6 (54.1)	174.9 (46.5)
HDL cholesterol (mg/dl)	55.4 (16.9)	54.6 (15.6)
Non-HDL cholesterol (mg/dl)	155.1 (51.1)	120.3 (43.2)
LDL-cholesterol (mg/dl)	127.1 (46.7)	95.7 (39)
Triglycerides (mg/dl)	160.1 (160.1)	133.4 (85)
GOT (UI/l)	—	27.3 (16.1)
GPT (UI/l)	—	27 (16.7)
GGT (UI/l)	—	40 (55.4)
Creatinine (mg/dl)	—	5.9 (20.4)
eGFR (CKD EPI) (ml/min/1.73 m^2^)	—	79.4 (16.3)
CK (mg/dl)	—	83.1 (62.9)

## Data Availability

Data are contained in the article.
